# Optimal Eukaryotic 18S and Universal 16S/18S Ribosomal RNA Primers and Their Application in a Study of Symbiosis

**DOI:** 10.1371/journal.pone.0090053

**Published:** 2014-03-03

**Authors:** Yong Wang, Ren Mao Tian, Zhao Ming Gao, Salim Bougouffa, Pei-Yuan Qian

**Affiliations:** 1 Division of Life Sciences, Hong Kong University of Science and Technology, Clear Water Bay, Hong Kong; 2 Sanya Institute of Deep Sea Science and Engineering, Chinese Academy of Sciences, San Ya, Hai Nan, China; University of Aveiro, Portugal

## Abstract

Eukaryotic 18S ribosomal RNA (rRNA) gene primers that feature a wide coverage are critical in detecting the composition of eukaryotic microscopic organisms in ecosystems. Here, we predicted 18S rRNA primers based on consecutive conserved sites and evaluated their coverage efficiency and scope of application to different eukaryotic groups. After evaluation, eight of them were considered as qualified 18S primers based on coverage rate. Next, we examined common conserved regions in prokaryotic 16S and eukaryotic 18S rRNA sequences to design 16S/18S universal primers. Three 16S/18S candidate primers, U515, U1390 and U1492, were then considered to be suitable for simultaneous amplification of the rRNA sequences in three domains. Eukaryotic 18S and prokaryotic 16S rRNA genes in a sponge were amplified simultaneously using universal primers U515 and U1390, and the subsequent sorting of pyrosequenced reads revealed some distinctive communities in different parts of the sample. The real difference in biodiversity between prokaryotic and eukaryotic symbionts could be discerned as the dissimilarity between OTUs was increased from 0.005 to 0.1. A network of the communities in external and internal parts of the sponge illustrated the co-variation of some unique microbes in certain parts of the sponge, suggesting that the universal primers are useful in simultaneous detection of prokaryotic and eukaryotic microbial communities.

## Introduction

Eukaryotic microbes play important roles as organic degraders, predators, producers and parasites [1]–[3]; however, our knowledge regarding their taxonomy, evolution, ecology and diversity remains limited. Eukaryotic microbes are typically ignored in most ecological settings, perhaps due to technical difficulties and a poor understanding of the microscopic world at this scale [4], [5]. As a result, a vital position for them in a variety of ecosystems has not been demonstrated. In some biospheres, there may be interplay between eukaryotic microbes and prokaryotic organisms [6], potentially mediating bioremediation and nutrient flow from autotrophic microbes to higher eukaryotes [7]. However, only prokaryotes have been reported in extreme environments; most studies have neglected to examine microbial eukaryotes [8], [9]. To what extent these eukaryotic microbes can spread into extreme conditions and the factors that are most critical to their survival are questions that need to be answered. The effects of anoxic conditions on protistan microbial communities were recently examined using 18S ribosomal DNA (rDNA) cloning and next-generation pyrosequencing techniques [10]–[12]. We have thus obtained a glimpse of the eukaryotic microbial communities present in environmental samples despite the difficulties encountered during sampling and data analysis [13]. However, eukaryotes have not been studied as extensively as prokaryotes in similar niches. For example, protist diversity was found to be much higher than originally estimated; it is believed that less than 10% of the rDNA sequences have been detected [14]. Despite a number of recent reports on biodiversity of eukaryotic microbes [15]–[17], their fitness to the local environment and role in the ecosystems are not determined. Thus, estimates of species richness and diversity of microbial eukaryotes and their relationship with prokaryotic organisms are a high priority of current studies. The relationships between eukaryotes and prokaryotes can be inferred from co-variation of the corresponding species. Therefore, efficient, simultaneous amplification of 16S and 18S rDNA sequences from a given sample is critical.

Small subunit ribosomal RNA (SSU rRNA) genes are the standard reference sequences for taxonomic classification of organisms. By calculating the similarity between rRNAs, Archaea was separated from Bacteria as an independent domain in 1977 [18]. Eukaryotic 18S rRNA primers have been applied in many studies investigating environmental communities [13], [19], [20]. In 2001, novel small eukaryotes were reported in an aphotic zone in the Antarctic polar front [21]. In addition, novel phylogenetic groups of fungal microbes have been defined using the same method [22]. Fungal diversity in indoor environments has been assessed using 18S rRNA amplicon pyrosequencing [23]. The eukaryotic primers A and B were designed in the 1980s and are still widely used at present [24]. Subsequently, several other eukaryote-specific and universal primers were developed, such as E82, E528, U1391 and U1492 [21], [25]. Although the 18S rRNA amplicon pyrosequencing technique has been applied to study environmental microbial eukaryotic communities, the specificity and coverage of the primers have not been evaluated. The addition of more eukaryotic rRNA sequences to the databases has revealed polymorphisms in several primer target regions, suggesting that significant eukaryotic diversity may be escaping detection. Thus, there are currently questions regarding the validity of some primers; optimization is required by modifying the primers to cover the most conserved regions. The number of 18S rRNA sequences in the current SILVA rRNA database is increasing rapidly [26]. In the present study, we used sequences available in this database to predict suitable primers for eukaryotic 18S rRNA genes and assess their coverage. The prediction scheme used herein to predict and evaluate prokaryotic primers has been introduced in our previous work [27]. Eighteen candidate eukaryotic primers were designed using the same protocol coupled with the ARB package [28]. These primers were evaluated together with a list of published 18S primers. Next, common conserved regions between the 16S and 18S rRNA genes were aligned and used to search for 16S/18S universal primers. A pair of 16S/18S universal primers was then applied to analyze different portions of a sponge sample. Co-variation of eukaryotic and prokaryotic organisms was obvious in some portions and, on the other hand, a heterogeneous distribution of a special group of bacteria was revealed in one of the sponge portions.

## Materials and Methods

### Prediction of candidate primers

Core aligned rDNA sequences were obtained from the database associated with the QIIME package (version 1.2) [29]. The following protocol was followed to generate the core rDNA sequences using QIIME. The original data were retrieved from the SILVA database (version 108) [26]. First, the sequences were sorted into OTUs at 3% dissimilarity, and then, representative sequences in each OTU were collected. The dataset of representatives was clustered using UCLUST into groups with no more than 80% identity. Those representatives from mitochondria and chloroplasts were filtered. These non-redundant rDNA sequences contained 747 eukaryotic, 1845 bacterial and 339 archaeal sequences. In addition, a full set of rDNA sequences was downloaded from SILVA (version 111) in both aligned and unaligned formats. Full-length rDNA sequences in the aligned format were selected from this version.

First, using the 747 aligned eukaryotic non-redundant sequences, candidate primers were designed using the tool provided in the ARB package [28]. Using default settings (primer length: 18 nt; minimum coverage: 50%), a list of candidate primers was obtained. The primers were positioned in reference to an 18S rDNA sequence from *Saccharomyces cerevisiae* (Z75578). Second, we searched for candidate primers using the protocol described in our previous work [27]. Briefly, we firstly located conserved sites in the aligned, non-redundant rDNAs. As described previously [27], conserved sites can be found in aligned positions in which a nucleotide is shared among >90% of the sequences. In cases in which a conserved position was occupied by two or even three principle nucleotides among >90% of the sequences, such a polymorphism was converted to a degeneracy. In general, a conserved region contains more than 15 consecutive conserved sites. Secondly, within the conserved regions, candidate primers were selected based on criteria of <5 degeneracies and longer than 15 nt.

### Examination of candidate primers

We tested the coverage efficiency of the candidate primers designed using ARB and our protocol. Full-length rDNAs in unaligned format served as targets for the selection of candidates. If a primer was identical or nearly identical to a part in an rDNA sequence, it was regarded as a suitable primer. To search for a matched region, a non-degenerate tetranucleotide from a primer was used as a seed. The seed in an rDNA sequence was extended at both ends to compare the sites between the primer and rDNA until two continuous mismatches were encountered. Degenerate sites in the primer were converted into polymorphisms, and all of the nucleotides in these sites were compared with the target sequentially. The tetranucleotide could be selected from the first and last four positions of the primer in that order of priority. If neither of the seeds could be used to find a target, we selected a non-degenerate tetranucleotide positioned in the middle of the primers. In this case, matching was extended at two directions of the landing site. If more than 15 matching sites and no more than two mismatches were recorded during seed landing and extension, the candidate primer was considered suitable for the rDNA. After using the primer to search all of the full-length rDNA sequences, the coverage rate was calculated as the percentage of rDNA sequences for which there was a primer match. Unmatched sequences were used to check for affiliated phyla. The specificity of a given primer could be summarized based on the taxonomic affiliations of the unmatched sequences.

### Pyrosequencing of 16S/18S rRNA genes from sponge samples

Sponge is a symbiotic system that contains both eukaryotic and prokaryotic microbes. It is, therefore, an ideal organism to examine amplification efficiency of the 16S/18S rDNA universal primers. A sponge sample was collected from a cold seep site (depth: ≈850 m) located at (22°17′N, 38°53′E). No specific permissions were required for these locations/activities. The field studies did not involve endangered or protected species and provide the specific location of the study at the Thuwal Seeps. The sponge was dissected into small pieces using sterile stainless steel scissors and then homogenized with a sterilized plastic mortar and pestle. Two samples from both the external (surface of sponge) (E1 and E2) and the internal (I1 and I2) parts of the sponge were used for the experiments. The homogenate was centrifuged (100× g, 1 min) to pellet debris and fragments of the sponge skeleton. The supernatant was then centrifuged at 10,000× g for 5 min, and the pellet was stored in 800 µL of DNA extraction buffer (500 mM NaCl, 50 Mm Tris-HCl (pH 8), 40 Mm EDTA, 0.75 M sucrose). DNA was extracted according to a previously reported protocol [30]. Briefly, cells were lysed in 10 µL of lysozyme (100 mg/mL), and then protein was degraded using 80 µL of 20% SDS and 8 µL of proteinase K (10 µg/µL). DNA was extracted twice using chloroform∶isoamyl alcohol (24∶1) and then precipitated with 0.6 vol of 100% isopropanol, followed by washing with 75% ethanol. The quality and quantity of the DNA were assessed using a NanoDrop ND-100 device (Thermo Fisher, USA) and agarose gel electrophoresis. The 16S/18S rRNA genes were amplified using universal primers (U515F and U1390R, designed in the present study) with barcodes. Each 20 µL PCR reaction consisted of 4 µL of 5× Phusion HF Buffer (M0530S, New England BioLabs, Inc.), 1.6 µL of dNTPs (2.5 µM each), 1 µL of forward and reverse primer (10 µM), 0.6 µL of DMSO, 10 ng of template DNA, 0.2 µL of DNA Polymerase (0.4 units) and 10.6 µL of ddH_2_O water. PCR was performed with a thermal cycler (Bio-Rad, USA) using the following program: an initial denaturation at 98°C for 1 min; 25 cycles at 98°C for 10 s, 56°C for 30 s and 72°C for 20 s; and a final extension at 72°C for 5 min. The concentration of each PCR amplicon was estimated by agarose gel electrophoresis, and an equal amount of each amplicon was mixed and pyrosequenced using a ROCHE 454 Titanium platform. Sequence data were deposited in NCBI (SRA) under accession no. SRX272400.

The microbial communities in I1 and E1 were examined again using eukaryotic and prokaryotic specific primers separately. The two samples were used to obtain 16S amplicons by using prokaryotic universal primers 905F (5′-TGAAACTYAAAGGAATTG-3′) (with modifications in reference to [27]) and 1492R (5′- GGTTACCTTGTTACGACTT-3′) [31]; and 18S amplicons by using eukaryotic primers 1A (5′- AACCTGGTTGATCCTGCCAGT-3′) [24] and 564R (5′- GGCACCAGACTTGCCCTC-3′) (with modifications in reference to [32]). PCR conditions were modified: for 16S amplification, an initial denaturation at 94°C for 5 min, followed by 25 cycles at 94°C for 50 s, 53°C for 50 s and 72°C for 50 s; for 18S amplification, an initial denaturation at 94°C for 5 min, followed by 25 cycles at 94°C for 50 s, 59°C for 50 s and 72°C for 50 s. I2 and E2 were excluded from the comparison to achieve deeper pyrosequencing of prokaryotic and non-sponge eukaryotic amplicons and better resolution of the communities of I1 and E1 in barcoded DNA samples.

### Analysis of pyrosequencing data

The pyrosequencing datasets were analyzed using the QIIME pipeline [29]. The sequences were filtered using quality controls as described previously [33]. Clusters in the amplicon reads were identified using UCLUST in QIIME. OTUs were collected at different dissimilarity levels (0.005–0.5). At a dissimilarity level of 3%, representative OTU reads were classified in reference to the SILVA database (version 111) with a threshold confidence level of 50%. A network of the four samples was created by make_otu_network.py in QIIME package. The output files containing nodes and edges were used to display the network of genera using Cytoscape [34]. Because the pyrosequencing platform could not obtain the full length of the amplicons, communities in the samples were retrieved from the reads for 5′ and 3′ ends of the amplicons, respectively.

## Results

### Optimal 18S ribosomal primers

Using the ARB package, five primers were predicted based on a core group of rDNA sequences from the SILVA database. Our pipeline differs from the ARB because consecutive, conserved sites are searched for initially. Within the conserved regions, the optimal primers were then selected. Using our pipeline, seven conserved regions were discovered (Table 1). Three regions overlapped with the primers predicted by ARB, and two were adjacent to ARB primers. Candidate primers for the region between 1624 nt and 1647 nt were not identified by our pipeline, whereas no primers were detected by ARB in the longest conserved region between 353 nt and 445 nt. The validity of the primers could be evaluated according to their coverage rates using the original core rDNA sequences. The coverage rates of ARB primers and the candidate primers in the conservative regions were calculated (Table 1). The values were generally larger than 80%. The lower values resulted from short sequences for which the primers did not have a matching target. For primers at both ends of the 18S rDNAs, conserved sites were detected by calculating the nucleotide frequencies in the full-length core sequences (Table S1). Candidate primers were selected to match positions 6–23 nt and 1755–1772 nt at the 5′ and 3′ ends, respectively (in reference to the *S. cerevisiae* 18S rRNA sequence). Together with the other candidates, a total of 18 primers were subjected to further assessment.

**Table 1:** Conserved regions in 18S eukaryotic rDNA and coverage percentages of predicted primers.

position	conserved region	% average primer coverage (SD)
ARB		
547–589	TGGAGGGCAAGTCTGGTGCCAGCAGCCGCGGTAATTCCAGCTC	93.3 (1.0)
1141–1161	GAATTGACGGAAGGGCACCAC	87.2 (0.7)
1266–1290	TGGTGGTGCATGGCCGTTCTTAGTT	85.9 (4.4)
1423–1443	TAACAGGTCTGTGATGCCCTT	86.3 (2.3)
1624–1647	CCTTTGTACACACCGCCCGTCGCT	82.4 (0.4)
this study		
353–445	ACGGGTRACGGRGRATYAGGGTTYGAYTCCGGAGAGGGA GCMTGAGARAYGGCTACYACWTCYAAGGAWGGCAGCAG GCRCGAAMTTRCCCA	82.6 (5.9)
549–589	GAGGGCAAGTCTGGTGCCAGCAGCCGCGGTAATTCCAGCTC	93.6 (0.9)
1181–1204	TTAATTTGACTCAACRCGGGRGAA	87.5 (2.5)
1266–1286	TGGTGGTGCATGGCCGTTCTT	88.9 (1.4)
1422–1440	ATAACAGGTCTGTGATGCC	85.9 (2.8)
1453–1471	GGGCYGCACGCGYRCTACA	78.7 (3.8)
1569–1586	AACGAGGAATKCCYWGTA	61.3 (2.2)

Conserved regions were identified using ARB software and a tool designed in this study. Potential primers were selected based on a sliding window of 16 nt. The coverage rates of these primers were averaged, and standard deviations (SD) were provided. The positions of the regions are in reference to the 18S rDNA sequence for *Saccharomyces cerevisiae* (Z75578). R: [A/G]; M: [A/C]; W[A/T]; K:[G/T];Y:[C/T].

First, 13105 full-length non-redundant eukaryotic 18S rDNA sequences were identified from the SILVA database together with sequences with a complete 5′ (1962) or 3′ (23752) end. These sequences were used to calculate the coverage rate for each candidate primer. For primers designed to recognize the 5′ end of the rDNA sequences, the evaluation was conducted utilizing the full-length non-redundant sequences and those with a complete 5′ end (Table 2). Overall, high coverage rates were achieved, but the two primers located at 353–374 nt and 370–389 nt performed poorly compared with the neighboring primer at 381–399 nt, which detected its target with high efficiency. This finding indicated that the sliding window approach over the conserved region permits the detection of more optimal primers. For the primers at the 3′ end, all sequences with a complete 3′ end were used for examination. The coverage rates were 95% or above for primers located at positions between 1141 nt and 1772 nt.

**Table 2:** Evaluation of candidate primers for eukaryotic 18S rDNA sequences.

Position	Sequence	% Coverage rate
6–23	GGTTGATYCTGCCAGTAG	94.7
353–374	ACGGGTRACGGRGRATYAGGGT	92.3
370–389	AGGGTTYGAYTCCGGAGAGG	91.5
381–399	TCCGGAGAGGGAGCMTGAGA	98.6
416–435	AAGGAWGGCAGCAGGCRCGA	97.6
422–445	GGCAGCAGGCRCGAAMTTRCCCA	96.5
549–566	GAGGGCAAGTCTGGTGCC	98.4
562–579	GTGCCAGCAGCCGCGGTA	98.5
573–589	CGCGGTAATTCCAGCTC	98.0
1141–1161	GAATTGACGGAAGGGCACCAC	97.4
1181–1204	TTAATTTGACTCAACRCGGG	98.7
1266–1286	TGGTGGTGCATGGCCGTTCTT	98.4
1422–1440	ATAACAGGTCTGTGATGCC	98.8
1453–1471	GGGCYGCACGCGYRCTACA	97.1
1569–1586	AACGAGGAATKCCYWGTA	95.7
1624–1642	CCTTTGTACACACCGCCCG	98.9
1629–1647	GTACACACCGCCCGTCGCT	98.3
1755–1772	AAGTCGTAACAAGGTWKC	96.2

Degeneracies and positions are indicated in Table 1.

**Figure 1:**
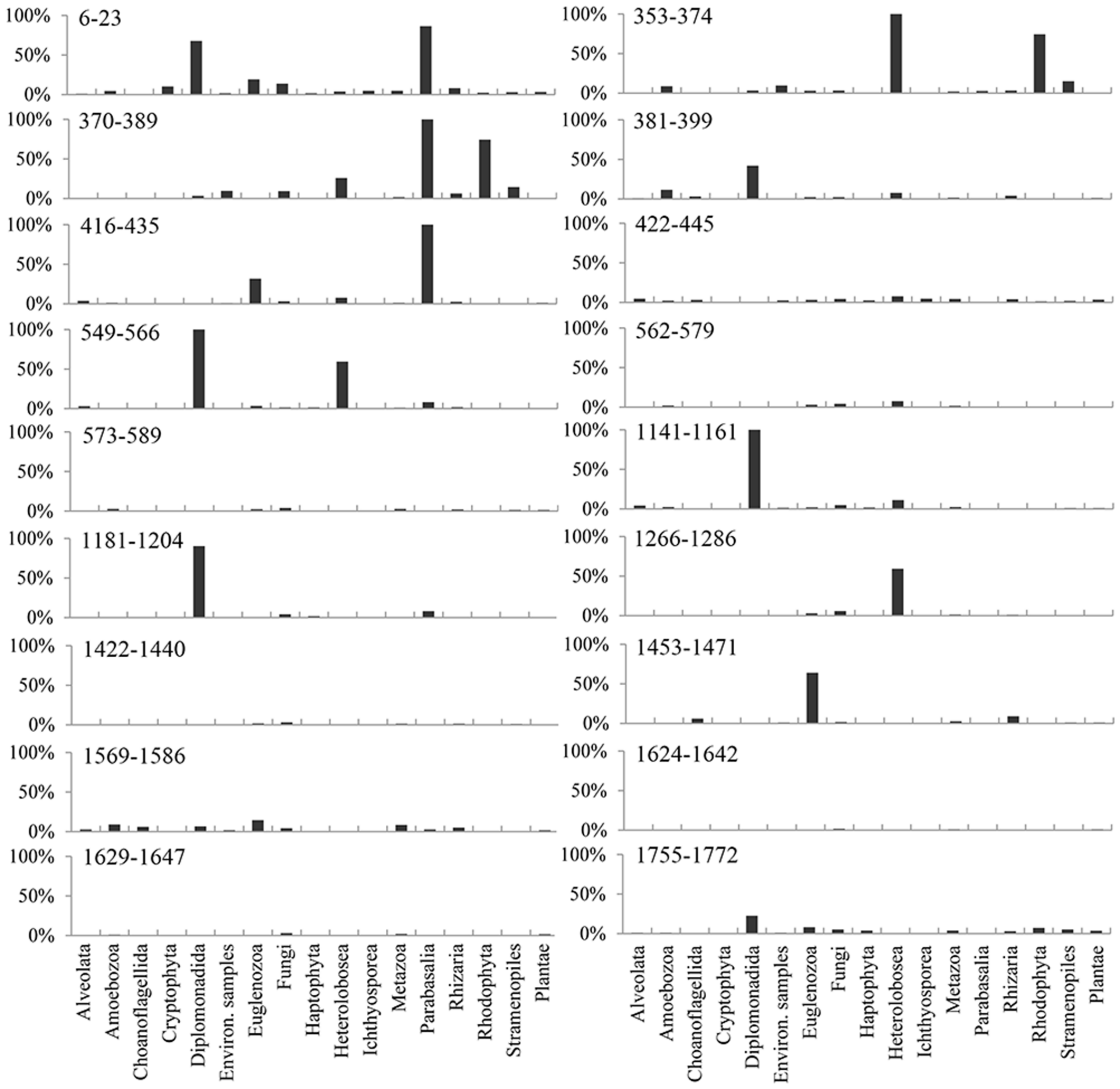
Failure detection rates for 18 candidate primers from different eukaryotic groups. The positions of the primers on the 18S rRNA gene (Z75578) from *S. cerevisiae* are indicated. Details and failure detection rates for the eukaryotic groups are listed in Table S2.

Next, we examined the performance of the primers applied to different phyla. Sequences that were not detected by the primers were sorted into different phyla or the highest levels following domain in the SILVA database. Some primers occasionally failed to detect a target in the majority of sequences from a particular phylum (Fig. 1). Both Euk353–374 and Euk370–389 (primer names indicate positions in *S. cerevisiae*) performed poorly against sequences from two phyla, consistent with their relatively low coverage rates (Table 2). Eight primers showed excellent coverage for a whole spectrum of eukaryotic taxa (Fig. 1). Among these primers, Euk1422–1440, Euk1624–1642 and Euk1629–1647 failed to detect, on average, only 0.4–0.6% of the reference sequences from the different phyla. Moreover, the sequences from Diplomonadida and Parabasalia were detected infrequently by these primers. The former had failure detection rates of >90% for the three primers; the latter was entirely undetectable by two of the primers in this *in silico* examination. Heterolobosea was more easily detected by the primers than Diplomonadida and Parabasalia, although a considerable percentage of its sequences were undetectable using several primers (Fig. 1).

### Evaluation of published primers

Several eukaryotic primers have been reported and applied to eukaryotic microbial studies. In addition, the coverage of these primers has been examined. Ten primers and their coverage rates are shown in Table 3. EukA (starts at position 1) and Euk4 are located at the 5′ end of the 18S rRNA genes and close to Euk6–23 developed in the present study. As indicated by the coverage rate, Euk6–23 outperformed EukA and Euk4 by a small margin despite significant overlap. Unlike EukA and Euk4, degenerate bases were introduced into Euk6–23. Therefore, the optimal primer targeting the 5′ end of 18S rDNA genes is Euk6–23 (see also Table S1).

**Table 3:** Coverage rate of published eukaryotic primers.

Primer	Position	Sequence	Coverage	Ref.
EukA	1–21	AACCTGGTTGATCCTGCCAGT	91.3%	[24]
Euk4	4–20	CTGGTTGATCCTGCCAG	89.8%	[24]
Euk82	83–99	GAAACTGCGAATGGCTC	89.3%	[21]
Euk360	382–399	CGGAGARGGMGCMTGAGA	98.4%	[24]
Euk516	548–563	GGAGGGCAAGTCTGGT	96.8%	[32]
Euk528	575–590	CGGTAATTCCAGCTCC	97.5%	[47]
Euk690	896–916	TCAGAGGTGAAATTCTTGGAT	94.3%	[48]
Euk1209	1426–1441	CAGGTCTGTGATGCCC	98%	[49]
U1391	1623–1641	CYCYTTGTACACACCGCCC	98.6%	[50]
U1492	1755–1774	AAGTCGTAACAAGGTAGCCGT	91.6%	[47]

The primer sequences are positioned on the 18S rDNA sequence for *S. cerevisiae* (Z75578).

No equivalent of Euk82 could be detected in the current study, perhaps due to its somewhat less satisfactory coverage rate of 89.3%. Euk360, which was associated with a high coverage rate, overlapped with our primers Euk370–389 and Euk381–399, and thus, it is also recommended. Euk516 and Euk528 are similar to primers developed in this study (Table 2); both successfully detected >96% of the dataset. Euk690 was not recognized by the ARB or by our pipeline; however, it still had a remarkably high coverage of 94.7%. Bacterial 16S rDNAs have a candidate primer in the same region, but its detection efficiency is low [27]. Euk1209, U1391 and U1492 were also recognized in the present study, albeit with minor positional variations. The former two primers covered a high percentage of the reference sequences; the latter produced a relatively lower coverage rate, likely due to its numerous mismatches caused by additional bases at the 3′ end and the absence of degenerate sites (Table S1).

### Comparison of 16S and 18S primers at equivalent functional regions

**Figure 2:**
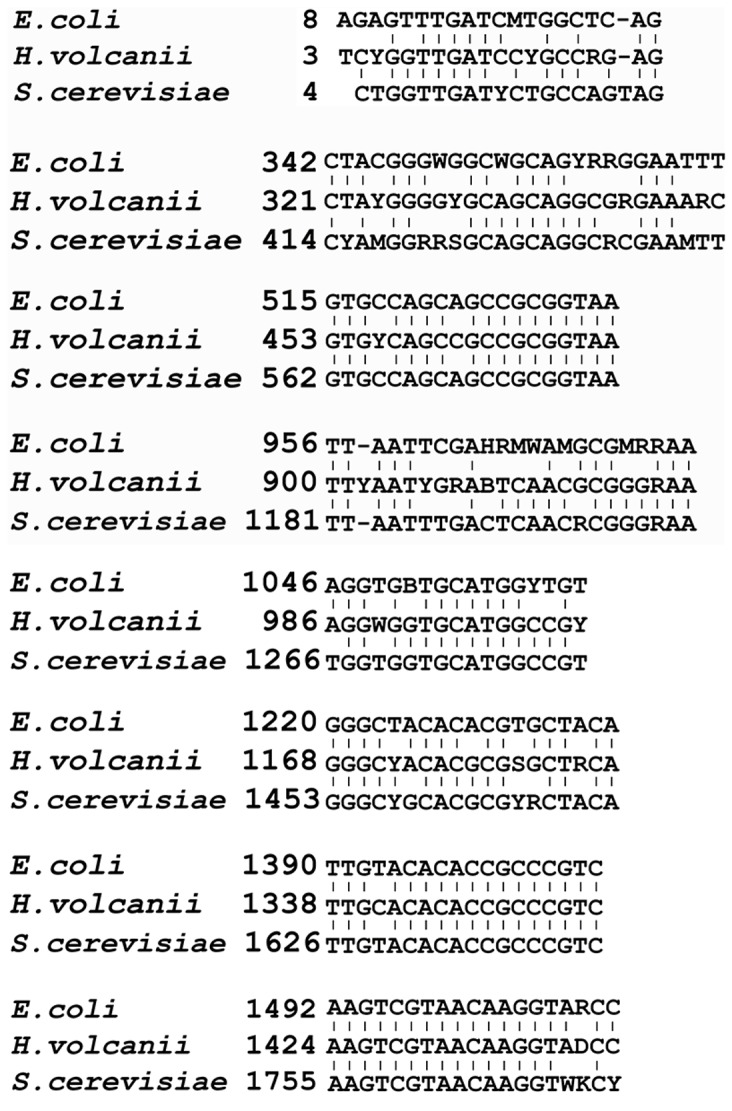
Alignment of common conserved regions in 16S and 18S rDNA sequences. The positions of the conserved regions are in reference to the 16S or 18S sequence from *Escherichia coli* MG1655 (U00006), *Halobacterium volcanii* (K00421) or *S. cerevisiae* (Z75578). Degenerate sites are indicated in Table 1.

In our previous work, 16S primers were predicted using the same protocol [27]. Here, prokaryotic primers were compared with the eukaryotic primers described above. This comparison may allow us to design universal primers for use in environmental microbial studies. Figure 2 shows the alignment of eight common conserved regions in SSU ribosomal genes across three domains: Archaea, Bacteria and Eukaryota. The remaining primers did not possess such long homologous regions in the prokaryotic sequences. In particular, the 18S primers Euk1422–1440 and Euk1569–1586 do not have prokaryotic counterparts, making them potentially useful for specific amplification of the eukaryotic rRNA genes. Euk1453–1471, in contrast, could be aligned with prokaryotic primers (Fig. 2). Therefore, Euk1422–1440 and Euk1569–1586 may be used to specifically amplify eukaryotic rRNA genes.

For the conserved region at 5′ end of the SSU rRNA genes, alignment of the consensus sequences from the three domains was not perfect; mismatches and indels (insertions and deletions) were observed (Fig. 2). Therefore, this region was considered not ideal for universal primer design. Here, we evaluated primers for Archaea (5′-GGTTGATCCYGCCRGAG-3′) and Bacteria (5′-AGAGTTTGATCMTGGCTCAG-3′), the coverage efficiency of which has not been tested previously. The results showed that both primers achieved 92.9% coverage for the 5′ complete sequences (228150 bacterial sequences; 9606 archaeal sequences).

Of seven downstream regions identified, the alignments starting at positions 342 and 956 of the *E. coli* sequence displayed more indels and mismatches than the 5′ end alignment (Fig. 2). Of the remaining five regions, four are possible targets for 16S/18S universal primers. The region beginning at position 1220 nt in *E. coli* had five degeneracies in the candidate primer and was not considered further. Thus, four universal primers were generated and applied to evaluate the coverage efficiency using full-length, non-redundant SSU rDNA sequences (13105 eukaryotic sequences; 124740 bacterial sequences; 2200 archaeal sequences) (Table 4). Excluding the primer U1047–1074 at positions 1047–1074 nt in *E. coli*, the primers featured high coverage rates (>95%) for the three domains. The poor performance of the primer U1047–1074 was highlighted by its coverage rate of 89.9% for archaeal sequences. When the full-length core sequences were tested, all of the primers except U1047–1074 showed a high coverage (>90%) for the different domains. Deterioration in coverage was mainly ascribed to mismatches in the eukaryotic sequences, ranging between 4% and 6% of the total sequences (Table 3). In contrast, the coverage decreased by, on average, 2.5% for prokaryotes, despite the non-redundancy imposed in the reference sequences. The coverage rate for the U1047–1074 was much lower when full-length, non-redundant sequences were applied, demonstrating a coverage rate of only 78.4% and 78.3% for Archaea and Bacteria, respectively. Although four degeneracies were introduced into the U1047–1074 primer, the observed coverage still deteriorated dramatically for the prokaryotic 16S core rDNA sequences. Therefore, it was considered unstable as a 16S/18S universal primer.

**Table 4:** Coverage percentage of universal primers for 16S/18S rRNA genes.

		Non-redundant			Core		
Position	Sequence	E	B	A	E	B	A
515–533	GTGYCAGCMGCCGCGGTAA	98.5	99.1	99.2	92.5	96.3	98.6
1047–1074	GGWGBTGCATGGYYG	97.3	93.9	89.9	93.3	78.3	78.4
1390–1407	TTGYACACACCGCCCGTC	98.6	99.6	99.9	93.2	97.4	98.6
1492–1507	AAGTCGTAACAAGGTW	95.6	95.9	96.4	91.1	91.6	93.2

Degeneracies are indicated in Table 1. In addition, B = T, C or G. The positions were adjusted to the 16S rDNA sequence from *E. coli*. The SILVA non-redundant and core full-length sequences were used as reference sequences to calculate the coverage for Eukaryota (E), Bacteria (B) and Archaea (A).

### Application of 16S/18S universal primers to sponge samples

**Figure 3:**
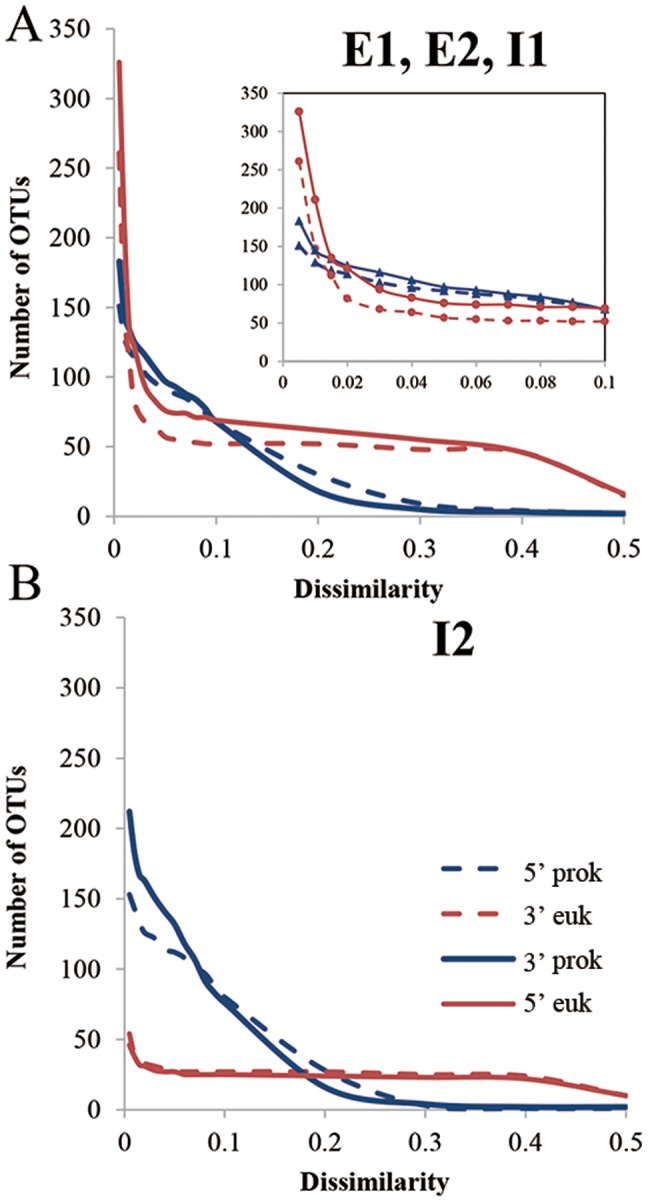
Number of OTUs at different dissimilarity levels. OTUs at different dissimilarity levels were counted for the 5′ and 3′ ends of the pyrosequenced amplicons. Prokaryotic (prok) and eukaryotic (euk) OTUs were counted separately. OTUs in samples E1, E2 and I1 (A) were summed, considering the high abundance of sponge 18S rDNA amplicons. The change of OTU numbers in the dissimilarity range of 0.005–0.1 is enlarged as shown in the inset (A). Sample I2 (B) is shown independently due to its high proportion of 16S rDNA amplicons.

To evaluate the 16S/18S universal primers, universal primers at positions of 515–533 and 1390–1407 in Table 4 were used to amplify DNA from external (surface) and internal sponge samples. SSU rDNA sequences from both prokaryotic and eukaryotic microbes were amplified in the same PCR reactions. Using gel electrophoresis, two clear bands of approximately 900 bp and 1300 bp in size were observed. The amplicons were pyrosequenced using a 454 platform, generating 25516 qualified reads. The reads were, on average, approximately 400 bp and unable to cover the whole amplicons. They were sorted into 9546 5′ reads and 15970 3′ reads. More operational taxonomic units (OTUs) were detected from the 3′ than from the 5′ ends at a dissimilarity of 3% (Fig. 3A), due to the larger number of qualified reads obtained for the 3′ end. SILVA classification of the 3′ reads of the amplicons revealed 24 eukaryotic and prokaryotic phyla (Table S3). Nevertheless, the communities as reflected by the major phyla were highly consistent for both ends of the amplicons. The results were compared with the microbial communities revealed by 16S and 18S specific primers using I1 and E1. With a cutoff of 0.1% in the community, a total of 11 phyla were detected by the two sets of primers. The major phyla were the same as those revealed by the 16S/18S primers. The 18S primers allowed to obtain Fungi, Metazoa and Crenarchaeota; the use of the 16S primers resulted in amplification of Metazoa, Proteobacteria and Crenarchaeota. Metazoa (sponge) amplicon reads accounted for a high proportion of the reads for the 16S and 18S specific amplicons. This was accounted for the insufficient pyrosequencing depth for the minority phyla, which may contribute to the differences in the composition of the communities revealed by the three sets of primers (Tables S3 and S4). Although Fungi were not identified in the 16S/18S amplicons for I1 and E1, they were present in the reads for I2 (0.09%). In addition, the percentages of Metazoa, Proteobacteria and Crenarchaeota in I1 and E1 were highly consistent in the reads for 16S/18S amplicons, but much differed in the reads for 16S-specific primers. Therefore, it seems that I1 had a surprisingly low proportion of sponge amplicon reads (55.6%) using the 16S-specific primers, compared with E1 (90%, close to the results of 16S/18S amplicon reads).

**Figure 4:**
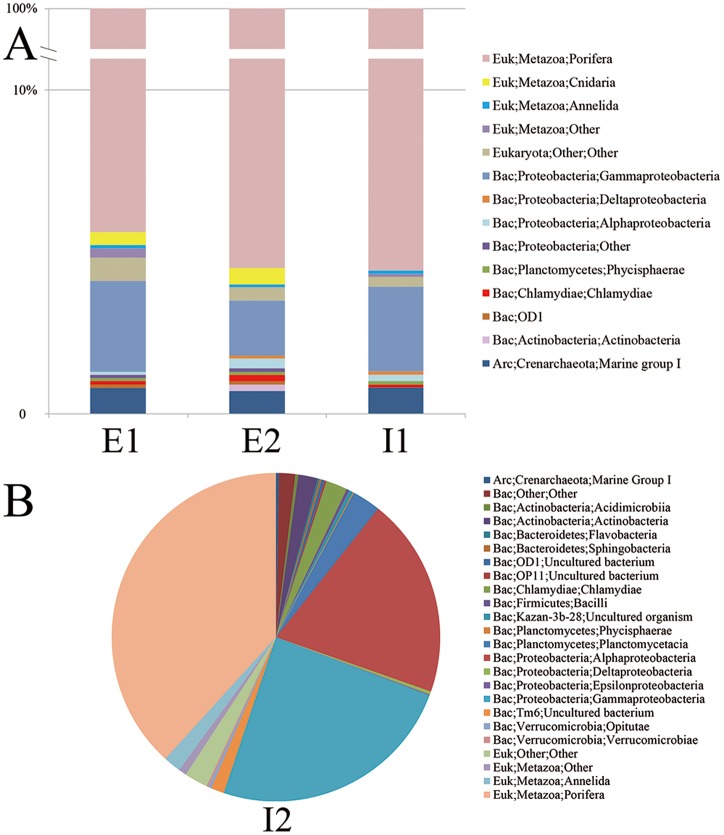
Taxonomic classification of 16S/18S amplicon reads. Sponge samples E1, E2 and I1 were highly represented by sponge amplicons (A), whereas the sample I2 had more bacterial than eukaryotic amplicon reads (B). The reads were classified into domains (Euk for Eukaryota; Bac for Bacteria; Arc for Archaea), phyla and classes in reference to SILVA database by RDP classifier with a threshold confidence level of 50%. The communities were based on the percentages of the reads for individual orders with a cutoff of 0.1%. The reads matched to the 5′ end of the amplicons were used for the classification.

Most of the reads (>90%) for E1, E2 and I1 belonged to a sponge species; only approximately 5% of them were sorted into other organisms that were dominated by Gammaproteobacteria (Fig. 4A). A considerable fraction of the eukaryotic reads were obtained via amplification of sponge 18S rDNAs; however, they could easily be filtered using a clustering method due to their high similarity. Unicellular eukaryotes were derived predominantly from the phylum Rhizaria (between 0.06% to 0.1% in I1, E1 and E2, see Table S3), which could be further sorted into the classes Foraminifera, Sticholonche and Acantharea. In the other internal sample I2, eukaryotes accounted for 43% of the total taxonomic read assignments (Fig. 4B), indicating that prokaryotes comprised the majority of the microbial community. Rhizaria, together with some eukaryotic environmental samples, were also not detected in I2. In contrast, I2 had some specific bacteria phyla, including Actinobacteria, Chlamydiae, Planctomycetes, Alphaproteobacteria and Tm6, all at least ten-fold higher than others. The assignment of the reads to the taxa revealed a high diversity of I2 as reflected by Shannon index of 1.3, compared to others with the index ranging between 0.06 and 0.12.

The amplicon reads were clustered into OTUs to reduce the representation of reads from identical eukaryotic genomes and sibling cells. This procedure permitted the observation of biodiversity at higher taxonomic levels. As expected, the number of OTUs for eukaryotes was much lower than that of their reads. At a dissimilarity of 0.005, the 14193 eukaryotic amplicons from samples E1, E2, and I1 formed just 250–300 OTUs in total, suggesting that they were highly similar and redundant. In contrast, there were 100–150 prokaryotic OTUs, which accounted for approximately 30% of the 16S amplicon reads. The eukaryotic OTU number decreased rapidly between the dissimilarity levels of 0.005–0.03 (Fig. 3A), which resulted in more prokaryotic than eukaryotic OTU numbers at 0.03 dissimilarity. At this dissimilarity, eukaryotic OTUs accounted for about 44.5% from the sponge samples E1, E2 and I1 (Table S5). Interestingly, the number of eukaryotic OTUs remained within a stable range of 50–70 until a dissimilarity of 40% was reached. This phenomenon is caused by the persistence of some singleton OTUs that are assigned to unknown eukaryotes in the datasets or probably resulted from false positive amplicons (Table S5). By comparison, the number of prokaryotic OTUs dropped steadily with increasing dissimilarity values. Eukaryotic reads for I2 were sorted into approximately 50 OTUs at 0.005 dissimilarity, comprising almost 11% of the associated number of amplicon reads. At dissimilarity levels below 0.18, there were more prokaryotic than eukaryotic OTUs (Fig. 3B). Therefore, prokaryotic species were obviously more enriched than eukaryotes in this internal sample compared with the other three samples. In addition, eukaryotic OTUs varied slightly in the dissimilarity range of 0.05–0.4, in agreement with the pattern shown in Fig. 3A.

The relationship of the genera in the four samples was illustrated in a network that was constructed based on nodes and edges at a dissimilarity level of 0.03 (Fig. S1). The most widely shared edges denoted co-existing microbes among the four samples and were highly represented by eukaryotes including the sponge. Archaeal microbes from Marine Group I (MGI) were abundant in I1 and E2 but were rarely observed in I2. Microbes that were shared between the internal samples comprised mainly Bacteria. Unlike the external samples and I1, almost all I2-specific species were derived from bacteria. Similarly, distribution of the eukaryotic microbes varied between different parts of the sponge. The colonization of some unknown eukaryotic organisms belonging to Cnidaria was probably restricted to the external samples (two OTUs and 34 amplicon reads for E1 and E2). Amplicons assigned to MGI Archaea were also detected in these samples. A greater number of reads were assigned to tubeworms (eggs, larvae and/or cells from dead bodies) similar to *Oligobrachia haakonmosbiensis* (Annelida) in I2 than in the other samples. I2 also had apparently more bacterial genera from *Rhizobium* (Alphaproteobacteria) *Halomonas* (Gammaproteobacteria) and *Shewanella* (Gammaproteobacteria). Therefore, a eukaryotic organism (Annelida) and several prokaryotic genera co-existed in such a unique microhabitat in the sponge.

## Discussion

In the present study, eukaryotic 18S rRNA primers were designated using the ARB package and our pipeline, aiming to study co-variation of prokaryotic and eukaryotic microbes in environments. The coverage rate and application spectrum of these candidate primers were also evaluated. This study provided a comprehensive survey of eukaryotic 18S primers that may facilitate further investigation of microscopic eukaryotic organisms in various habitats and in symbiotic systems. In addition, we examined the performance of published 18S primers in our pipeline. Overall, we confirmed that most of these primers remained efficient in studies of the eukaryotic community. However, some of the primers targeting the ends of the 18S rDNA sequences would probably fail to detect an unacceptably high percentage of eukaryotic species. The problematic regions in the primer design were identified by statistical analysis of the nucleotide frequencies at both ends of the sequences. Furthermore, the published primers evaluated herein contain insufficient degenerate sites, similar to those produced using the ARB package. The improved performance of the primers designed in the present study may be due to the accurate introduction of some degenerate sites. Therefore, the primers predicted by our pipeline should possess optimal detection efficiency.

Our pipeline also showed some weaknesses in the present study. For example, using our pipeline, we could not detect Euk82, Euk690 or Euk1624–1642; Euk82 and Euk690 have been used publicly, whereas Euk1624–1642 was obtained from the ARB package. The 18S rRNA region targeted by Euk690 was less conserved in prokaryotic rRNA genes [27], which may explain why this region was not detected by either the ARB package or our pipeline. Surprisingly, the coverage rate of Euk690 was almost 95% and the 16S primer positioned at the same region displayed a relatively low coverage rate [27]. This unexpected result is likely due to the length of the conserved region. Our match criteria allowed a maximum of two mismatches and a minimum of 15 matches, which probably enhanced the coverage of the 21 nt primer. Using the TestProbe tool in the SILVA database, the coverage rate of Euk690 for Eukaryota was merely 78% when two mismatches were allowed. This difference indicated that TestProbe counted all of the mismatches between the target region and the 21 nt primer Euk690. To test the effect of primer size, Euk690 was shortened to 16 nt and again checked with TestProbe. The coverage ranged between 92% and 94.5%, consistent with our results for Euk690. Additional evidence was obtained using primers ranging in size from 16 nt to 18 nt, for which TestProbe generated almost the same coverage as our pipeline. The failure in detecting Euk82 and Euk1624–1647 may be due to the inclusion of short sequences in the non-redundant core sequences because it was impossible to distinguish the real alignment gaps inside of those short sequences and the gap marks beyond the 3′ end. The same situation was encountered using the primers located at 5′ end of the sequences. Thus, full-length sequences were selected as targets in subsequent analyses of the coverage efficiency of candidate primers, which was based on 18S rDNA sequences from known taxonomic groups, and thus, the actual detection rate against as yet undiscovered eukaryotic groups could not be discerned. Hence, caution should be taken when using primers with the potential to overlook several eukaryotic groups. Such an effect may be amplified when two such primers are applied to the investigation of rare environmental samples [35].

In the present study, we compared 16S and 18S primers for the rRNA genes to search for universal rDNA primers. Common conserved sites in these rDNA primers are considered functionally important for ribosomes [36], [37]. Discrepancies between the 16S and 18S conserved regions might co-occur with direct genetic events responsible for evolutionary divergence among the organisms in the three taxonomic domains. Eukaryotic 18S rRNA genes are longer than prokaryotic 16S rRNA genes. The rRNA region that contributed to the expansion of 18S rRNAs in the ancestral sequences can be identified from the alignment of homologous primers. The distance between the primers differs between *S. cerevisiae* and the prokaryotic species (Fig. 2), whereas the largest insertions occurred in the region between 515 nt and 956 nt (in reference to the *E. coli* rRNA gene). In prokaryotes, this region is targeted by at least two prokaryotic universal 16S primers, Prok785 and Prok909, along with several bacterial and archaeal primers [27]. Because there are no other 18S primers for this region except Euk690, the prokaryotic rRNA genes could be amplified specifically using Prok785, Prok909 and several other primers.

The most conserved regions in the 16S/18S primers might be used to design universal primers that target both eukaryotes and prokaryotes. Such primers are required for the simultaneous detection of prokaryotic and eukaryotic organisms in a given environment. In a recent study, the prokaryotic 16S and eukaryotic 18S primers were used separately with the goal of exploring the co-occurrence of prokaryotic and eukaryotic microbes in rumens [15]. In that study, complex approaches were employed to determine the proportions of Archaea, Bacteria and Eukaryotes. Therefore, the three primers recommended in the present study should be highly valuable for the exploration of environments in which eukaryotic organisms and prokaryotic microbes interact to form a microscopic biological network. Application of these primers will permit investigation of the *in situ* species richness of eukaryotes and prokaryotic microbes simultaneously and at a lower cost. Alternatively, analyses of pyrosequenced metagenomes may provide similar insights; however, pyrosequencing of PCR amplicons is a more direct and efficient method. In the current study, microbial inhabitants in different parts of the sponge were documented. The co-existence and co-variation of eukaryotic and prokaryotic microbes likely provided an unprecedented view of relationships developed in the sponge. The validity of the 16S/18S universal primers was checked by comparing the pyrosequenced amplicons with those derived from 16S and 18S specific primers, separately. Results showed that the major community compositions in the sponge samples were highly consistent. However, one shortcoming of these primers was their tendency to generate false-positive PCR products when applied to eukaryotes, which might have resulted in an overestimation of eukaryotic OTUs (as shown in Fig. 3). This was also likely a result of adding barcodes to the primers. Representative reads in these OTUs were classified as unknown Eukaryota, but most of them were not 18S rDNA amplicons (unpublished results). The amplicon reads representing noise could have been removed by more stringent filtering criteria or verified using HMM-based rRNA identification tools such as Meta_RNA [38].

Two genetic factors are critical for the application of universal primers to evaluation of the covariation. The first is the copy number of SSU rRNA genes in a genome. As previously mentioned, the copy number of 16S genes in prokaryotic genomes is much less than that in eukaryotic genomes, which makes it difficult to determine the ratio of eukaryotic to prokaryotic cells in a sample. In general, the copy number of 18S rRNA genes is proportional to the size of the eukaryotic genome [39], [40]. In contrast, the prokaryotic 16S rRNA gene copy number ranges from one to several and is affected by the resource availability [41], [42]. Approximately 40% of bacterial genomes have one or two 16S rRNA genes that are almost identical [42], whereas the eukaryotic genome can have up to thousands of 18S rRNA genes [40]. A recent study reported that the sponge genome has an average size of 0.2 pg [43]. Given this size, the rRNA copy number in a sponge genome is approximately 100, according to the previous work by Prokopowich et al. [40]. Although we did not estimate the genome size of the sponge in the present study, the copy number of 18S rRNA genes for the sponge and other eukaryotic microbes is certainly higher than that of 16S rRNA genes for associated prokaryotes. The second factor is the genetic distance between SSU rRNA gene copies in sibling cells. Although this distance is theoretically higher than that between the intra-chromosomal copies, genetic polymorphisms among environmental eukaryotic microscopic organisms originating from the same stem cells remain unknown. The degree of polymorphism may depend on the environment [44]. In the present study, five sponge OTUs were detected in the four samples at 3% dissimilarity (Table S5). Therefore, the intra-chromosomal genetic polymorphisms could be easily quantified by grouping the amplicon pyrosequencing reads at a certain dissimilarity level; thus, the number of OTUs could be used to estimate the relative biodiversity between prokaryotes and eukaryotes.

The average size of the 454 pyrosequencing reads from the Titanium platform was approximately 400 bp, and thus was able to complete either the 5′ or the 3′ end of the amplicons, and consequently, the reads were separated into two groups. The 5′ amplicon reads contained the variant regions of the SSU rDNAs starting from V4, whereas the 3′ amplicon reads terminated at the V8 region [45]. Within the two groups, the number of qualified pyrosequencing reads differed; however, in the present study, their taxonomic classification revealed almost congruent communities. However, since the numerous sponge amplicons in the samples occupied a high percentage of the 454 pyrosequencing reads, a sequencing depth for amplicons from other species was insufficient, which suggests that 454 pyrosequencing is a costly method with low efficiency for similar pyrosequencing studies of 16S/18S amplicons. Nevertheless, as main objective of the present experiment was to examine universal primers, sequencing depth was not our major concern herein.

In this study, we successfully applied universal primers to study sponge-associated microbes and revealed their distribution heterogeneity in the sponge. Both eukaryotic and prokaryotic communities in different parts of a deep sea sponge species were examined simultaneously. As suggested above, it was not important to compare the absolute number of reads from eukaryotes and prokaryotes independently. Therefore, the communities, including the sponge host and all of the microbial inhabitants, were displayed by clustering the associated 16S/18S amplicon reads into different OTUs. The detection of Cnidaria and Annelida in the sponge samples was a surprising finding in the present study, because the presence of these phyla in sponge tissues has not been reported previously. In this study, the inner part of the sponge was enriched with nitrogen-fixing *Rhizobium spp.* together with the Annelida. The internal sample I1 might not be an ideal replicate of I2 because of its similarity to the external samples in terms of the abundance of eukaryotic cells. Thus, the I1 sample may have been rather close to the sponge surface, indicating the internal variations of microbiological habitats. The different roles of microbes in a sponge, such as obligate symbionts, opportunistic microbes or food might be indicated by their distribution and co-occurrence [46].

## Supporting Information

Figure S1 **Network of species present in sponge samples.** Samples from external (E1 and E2 nodes) and internal (I1 and I2 nodes) regions of the sponge were used to construct a network of microbial inhabitants. At a dissimilarity level of 3%, network data generated by QIIME using 3′ reads of the 16S/18S rDNA amplicons was used as input. Archaea (green), Bacteria (blue) and Eukaryotes (red) shared among the samples were connected by lines (edges for genera).
(TIF)

Table S1 **Statistics of nucleotide occurrence at 5′ and 3′ ends of 18S rDNAs.**
(DOCX)

Table S2 **Percentage of sequences failed to be matched by the candidate 18S primers.** In the first line, only the start position of the primers is shown. The full positions of the primers were labeled on Figure 1. Refer to Table 1 for the positions of the primers.
(DOCX)

Table S3 **Taxonomic classification at phylum level.** The statistics is based on taxonomic classification of the amplicon reads at 3′ end of the amplicons.
(DOCX)

Table S4 **Percentage of phyla revealed by 16S and 18S-specific primers.** 16S primers were 905F and 1492R; 18S primers were 1A and 564R. Percentages for three known classes of Metazoa are also shown.
(DOCX)

Table S5 **Statistics of OTUs using 3′ reads of the amplicons at 3% dissimilarity.** The number of singleton OTUs assigned to Eukaryota was over-estimated. Most of the reads in the singletons were a result of false positive amplifications by the universal primers.
(DOCX)
