# Supplementary figures and images for: Optimal Eukaryotic 18S and Universal 16S/18S Ribosomal RNA Primers and Their Application in a Study of Symbiosis
Source: PLoS One. 2014 Mar 3;9(3):e90053. doi: 10.1371/journal.pone.0090053 (PMC3940700; doi:10.1371/journal.pone.0090053)

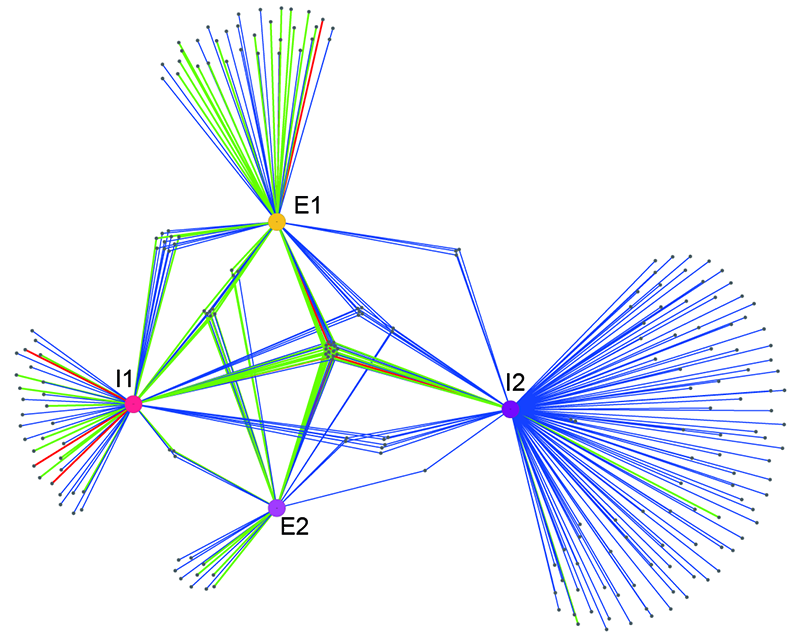

Supplement: Figure S1 — Network of species present in sponge samples. Samples from external (E1 and E2 nodes) and internal (I1 and I2 nodes) regions of the sponge were used to construct a network of microbial inhabitants. At a dissimilarity level of 3%, network data generated by QIIME using 3′ reads of the 16S/18S rDNA amplicons was used as input. Archaea (green), Bacteria (blue) and Eukaryotes (red) shared among the samples were connected by lines (edges for genera). (TIF) [file pone.0090053.s001.tif]
