# Supplementary material for: Optimal Eukaryotic 18S and Universal 16S/18S Ribosomal RNA Primers and Their Application in a Study of Symbiosis
Source: PLoS One. 2014 Mar 3;9(3):e90053. doi: 10.1371/journal.pone.0090053 (PMC3940700; doi:10.1371/journal.pone.0090053)
Supplement: Table S1 — Statistics of nucleotide occurrence at 5′ and 3′ ends of 18S rDNAs. (DOCX) [file pone.0090053.s002.docx]

Table S1 Statistics of nucleotide occurrence at 5’ and 3’ ends of 18S rDNAs

| 5’ | consensus | occurrence frequency | | | | 3’ | consensus | occurrence frequency | | | |  |
| --- | --- | --- | --- | --- | --- | --- | --- | --- | --- | --- | --- | --- |
| position | base | A | G | C | T | position | base | A | G | C | T | |
| 2 | A | 86% | 3% | 3% | 8% | 1755 | A | 98% | 1% | 0% | 1% | |
| 3 | Y | 7% | 3% | 78% | 13% | 1756 | A | 97% | 1% | 0% | 2% | |
| 4 | C | 1% | 1% | 95% | 2% | 1757 | G | 0% | 99% | 0% | 1% | |
| 5 | T | 7% | 0% | 5% | 88% | 1758 | T | 1% | 1% | 1% | 97% | |
| 6 | G | 1% | 97% | 1% | 1% | 1759 | C | 1% | 0% | 98% | 1% | |
| 7 | G | 3% | 94% | 1% | 1% | 1760 | G | 3% | 97% | 0% | 0% | |
| 8 | T | 1% | 2% | 1% | 95% | 1761 | T | 1% | 0% | 1% | 98% | |
| 9 | T | 1% | 2% | 1% | 96% | 1762 | A | 98% | 0% | 1% | 1% | |
| 10 | G | 2% | 95% | 1% | 2% | 1763 | A | 98% | 1% | 1% | 0% | |
| 11 | A | 96% | 1% | 3% | 0% | 1764 | C | 1% | 1% | 96% | 2% | |
| 12 | T | 1% | 1% | 1% | 97% | 1765 | A | 96% | 2% | 1% | 1% | |
| 13 | Y | 1% | 1% | 86% | 12% | 1766 | A | 95% | 2% | 2% | 1% | |
| 14 | C | 2% | 1% | 95% | 2% | 1767 | G | 1% | 99% | 0% | 0% | |
| 15 | T | 1% | 1% | 1% | 97% | 1768 | G | 1% | 95% | 1% | 3% | |
| 16 | G | 1% | 95% | 1% | 3% | 1769 | T | 1% | 1% | 3% | 95% | |
| 17 | C | 2% | 1% | 94% | 3% | 1770 | W | 20% | 3% | 4% | 73% | |
| 18 | C | 0% | 1% | 97% | 2% | 1771 | K | 9% | 12% | 3% | 76% | |
| 19 | A | 93% | 1% | 1% | 5% | 1772 | C | 1% | 1% | 90% | 8% | |
| 20 | G | 1% | 96% | 1% | 2% | 1773 | Y | 5% | 6% | 75% | 14% | |
| 21 | T | 4% | 3% | 2% | 91% | 1774 | G | 3% | 93% | 2% | 2% | |
| 22 | A | 88% | 6% | 3% | 3% | 1775 | T | 2% | 1% | 2% | 95% | |
| 23 | G | 8% | 87% | 3% | 2% | 1776 | A | 94% | 2% | 1% | 3% | |

See Table 1 for the positions of the primers.
