# Supplementary material for: Optimal Eukaryotic 18S and Universal 16S/18S Ribosomal RNA Primers and Their Application in a Study of Symbiosis
Source: PLoS One. 2014 Mar 3;9(3):e90053. doi: 10.1371/journal.pone.0090053 (PMC3940700; doi:10.1371/journal.pone.0090053)
Supplement: Table S2 — Percentage of sequences failed to be matched by the candidate 18S primers. In the first line, only the start position of the primers is shown. The full positions of the primers were labeled on Figure 1. Refer to Table 1 for the positions of the primers. (DOCX) [file pone.0090053.s003.docx]

Table S2 Percentage of sequences failed to be matched by the candidate 18S primers

| Taxo Level | Name | 6 | 353 | 370 | 381 | 416 | 422 | 549 | 562 | 573 | 1141 | 1181 | 1266 | 1422 | 1453 | 1569 | 1624 | 1629 | 1755 |
| --- | --- | --- | --- | --- | --- | --- | --- | --- | --- | --- | --- | --- | --- | --- | --- | --- | --- | --- | --- |
| superphylum | Alveolata | 1.0% | 0.6% | 0.4% | 0.8% | 3.5% | 4.3% | 2.9% | 0.7% | 1.0% | 4.0% | 0.3% | 0.5% | 0.4% | 0.6% | 2.6% | 0.8% | 0.8% | 1.2% |
| phylum | Amoebozoa | 4.3% | 8.6% | 0.5% | 11.2% | 1.1% | 2.1% | 0.5% | 2.1% | 2.7% | 2.1% | 0.0% | 0.0% | 0.0% | 0.0% | 9.1% | 0.5% | 1.1% | 1.1% |
| class | Choanoflagellida | 0.0% | 0.0% | 0.0% | 2.9% | 0.0% | 2.9% | 0.0% | 0.0% | 0.0% | 0.0% | 0.0% | 0.0% | 0.0% | 5.9% | 5.9% | 0.0% | 0.0% | 0.0% |
| phylum | Cryptophyta | 10.3% | 0.0% | 0.0% | 0.0% | 0.0% | 0.0% | 0.0% | 0.0% | 0.0% | 0.0% | 0.0% | 0.0% | 0.0% | 0.0% | 0.0% | 0.0% | 0.0% | 0.0% |
| order | Diplomonadida | 67.7% | 3.2% | 3.2% | 41.9% | 0.0% | 0.0% | 100% | 0.0% | 0.0% | 100% | 90.3% | 0.0% | 0.0% | 0.0% | 6.5% | 0.0% | 0.0% | 22.6% |
| n/a | Environ. samples | 1.7% | 9.5% | 9.5% | 0.3% | 0.7% | 2.5% | 0.4% | 0.5% | 0.8% | 1.3% | 0.8% | 0.1% | 0.0% | 1.2% | 1.8% | 0.3% | 0.7% | 1.3% |
| phylum | Euglenozoa | 19.3% | 2.9% | 0.0% | 2.0% | 31.6% | 2.9% | 3.3% | 2.9% | 2.5% | 2.0% | 0.0% | 2.9% | 1.6% | 63.9% | 14.3% | 0.0% | 0.0% | 8.2% |
| kingdom | Fungi | 13.8% | 3.2% | 9.2% | 1.9% | 2.8% | 4.2% | 1.5% | 4.2% | 4.0% | 4.6% | 4.1% | 5.5% | 2.7% | 1.8% | 4.2% | 1.9% | 3.1% | 5.1% |
| phylum | Haptophyta | 1.5% | 0.8% | 0.0% | 0.0% | 0.0% | 2.3% | 1.5% | 0.8% | 0.0% | 1.5% | 1.5% | 0.0% | 0.8% | 0.8% | 0.0% | 0.0% | 0.0% | 3.8% |
| class | Heterolobosea | 3.7% | 100% | 25.9% | 7.4% | 7.4% | 7.4% | 59.3% | 7.4% | 0.0% | 11.1% | 0.0% | 59.3% | 0.0% | 0.0% | 0.0% | 0.0% | 0.0% | 0.0% |
| class | Ichthyosporea | 4.5% | 0.0% | 0.0% | 0.0% | 0.0% | 4.5% | 0.0% | 0.0% | 0.0% | 0.0% | 0.0% | 0.0% | 0.0% | 0.0% | 0.0% | 0.0% | 0.0% | 0.0% |
| kingdom | Metazoa | 4.7% | 1.8% | 1.7% | 1.3% | 1.1% | 4.1% | 1.0% | 1.8% | 2.7% | 2.4% | 0.4% | 1.3% | 1.5% | 2.6% | 8.5% | 1.2% | 2.2% | 3.8% |
| phylum | Parabasalia | 86.5% | 2.7% | 100% | 0.0% | 100 % | 0.0% | 8.1% | 0.0% | 0.0% | 0.0% | 8.1% | 0.0% | 0.0% | 0.0% | 2.7% | 0.0% | 0.0% | 0.0% |
| supergroup | Rhizaria | 8.1% | 3.0% | 6.3% | 3.8% | 2.3% | 3.8% | 1.8% | 1.0% | 2.0% | 0.5% | 0.5% | 1.0% | 1.3% | 8.8% | 5.1% | 0.8% | 0.5% | 3.0% |
| phylum | Rhodophyta | 2.1% | 74.4% | 74.2% | 0.1% | 0.4% | 1.3% | 0.3% | 0.4% | 0.8% | 0.3% | 0.1% | 0.4% | 0.3% | 0.0% | 0.3% | 0.0% | 0.0% | 7.3% |
| phylum | Stramenopiles | 2.9% | 14.9% | 14.4% | 0.4% | 0.4% | 1.8% | 0.3% | 0.4% | 1.5% | 1.0% | 0.9% | 0.8% | 1.0% | 1.1% | 1.0% | 0.2% | 0.9% | 5.1% |
| phylum | Plantae | 3.1% | 0.6% | 0.5% | 1.1% | 1.0% | 3.4% | 0.6% | 0.9% | 1.4% | 1.0% | 0.7% | 0.5% | 0.9% | 1.0% | 1.9% | 1.3% | 1.9% | 3.7% |

In the first line, only the start position of the primers is shown. The full positions of the primers were labeled on Figure 1. Refer to Table 1 for the positions of the primers.
