# Supplementary material for: Optimal Eukaryotic 18S and Universal 16S/18S Ribosomal RNA Primers and Their Application in a Study of Symbiosis
Source: PLoS One. 2014 Mar 3;9(3):e90053. doi: 10.1371/journal.pone.0090053 (PMC3940700; doi:10.1371/journal.pone.0090053)
Supplement: Table S3 — Taxonomic classification at phylum level. The statistics is based on taxonomic classification of the amplicon reads at 3′ end of the amplicons. (DOCX) [file pone.0090053.s004.docx]

Table S3 Taxonomic classification at phylum level

| Taxon | E1 | E2 | I1 | I2 |
| --- | --- | --- | --- | --- |
| Arc;Crenarchaeota | 0.82% | 0.67% | 0.75% | 0.35% |
| Arc;Euryarchaeota | 0.05% | 0.00% | 0.03% | 0.09% |
| Bac;Acidobacteria | 0.02% | 0.00% | 0.00% | 0.00% |
| Bac;Actinobacteria | 0.05% | 0.21% | 0.11% | 2.34% |
| Bac;Bacteroidetes | 0.02% | 0.05% | 0.06% | 0.35% |
| Bac;OD1 | 0.06% | 0.05% | 0.02% | 0.26% |
| Bac;OP11 | 0.00% | 0.00% | 0.00% | 0.17% |
| Bac;SR1 | 0.00% | 0.00% | 0.00% | 0.09% |
| Bac;Chlamydiae | 0.06% | 0.21% | 0.06% | 2.08% |
| Bac;Chloroflexi | 0.00% | 0.05% | 0.00% | 0.00% |
| Bac;Cyanobacteria | 0.00% | 0.00% | 0.02% | 0.00% |
| Bac;Firmicutes | 0.00% | 0.00% | 0.00% | 0.43% |
| Bac;Gemmatimonadetes | 0.02% | 0.00% | 0.00% | 0.00% |
| Bac;Kazan-3b-28 | 0.00% | 0.00% | 0.00% | 0.35% |
| Bac;Lentisphaerae | 0.03% | 0.00% | 0.00% | 0.00% |
| Bac;Planctomycetes | 0.09% | 0.05% | 0.14% | 2.86% |
| Bac;Proteobacteria | 2.90% | 2.00% | 2.80% | 44.10% |
| Bac;Spirochaetes | 0.03% | 0.00% | 0.03% | 0.00% |
| Bac;Tenericutes | 0.00% | 0.00% | 0.02% | 0.00% |
| Bac;Tm6 | 0.02% | 0.00% | 0.03% | 1.30% |
| Bac;Verrucomicrobia | 0.00% | 0.00% | 0.00% | 0.43% |
| Euk;Fungi | 0.00% | 0.00% | 0.00% | 0.09% |
| Euk;Metazoa | 94.92% | 96.12% | 95.28% | 40.78% |
| Euk;Rhizaria | 0.06% | 0.10% | 0.08% | 0.00% |
|  |  |  |  |  |
|  |  |  |  |  |
|  |  |  |  |  |

The statistics is based on taxonomic classification of the amplicon reads at 3’ end of the amplicons.
