# Supplementary material for: Optimal Eukaryotic 18S and Universal 16S/18S Ribosomal RNA Primers and Their Application in a Study of Symbiosis
Source: PLoS One. 2014 Mar 3;9(3):e90053. doi: 10.1371/journal.pone.0090053 (PMC3940700; doi:10.1371/journal.pone.0090053)
Supplement: Table S4 — Percentage of phyla revealed by 16S and 18S-specific primers. 16S primers were 905F and 1492R; 18S primers were 1A and 564R. Percentages for three known classes of Metazoa are also shown. (DOCX) [file pone.0090053.s005.docx]

Table S4 Percentage of phyla revealed by 16S and 18S-specific primers

|  |  |  |
| --- | --- | --- |
| 18S primers | I1 | E1 |
| Crenarchaeota | 0.2% | 0.1% |
| Eukaryota;Other | 0.1% | 0.0% |
| Fungi | 0.1% | 2.1% |
| Metazoa | 99.6% | 97.9% |
| Metazoa;Annelida | 0.0% | 0.1% |
| Metazoa;Cnidaria | 0.1% | 0.1% |
| Metazoa;Porifera | 99.4% | 97.6% |
| 16S primers |  |  |
| Crenarchaeota | 18.6% | 5.6% |
| Euryarchaeota | 0.1% | 0.0% |
| Actinobacteria | 0.1% | 0.0% |
| Bacteroidetes | 0.2% | 0.0% |
| Lentisphaerae | 0.1% | 0.0% |
| Planctomycetes | 0.8% | 0.0% |
| Proteobacteria | 22.8% | 3.2% |
| Rf3 | 0.1% | 0.0% |
| Tenericutes | 0.1% | 0.0% |
| Eukaryota;Other | 0.1% | 0.1% |
| Metazoa | 56.8% | 90.9% |
| Metazoa;Other | 0.1% | 0.1% |
| Metazoa;Cnidaria | 0.0% | 0.4% |
| Metazoa;Mollusca | 0.1% | 0.0% |
| Metazoa;Porifera | 55.6% | 90.3% |

16S primers were 905F and 1492R; 18S primers were 1A and 564R. Percentages for three known classes of Metazoa are also shown.
