# Supplementary material for: Optimal Eukaryotic 18S and Universal 16S/18S Ribosomal RNA Primers and Their Application in a Study of Symbiosis
Source: PLoS One. 2014 Mar 3;9(3):e90053. doi: 10.1371/journal.pone.0090053 (PMC3940700; doi:10.1371/journal.pone.0090053)
Supplement: Table S5 — Statistics of OTUs using 3′ reads of the amplicons at 3% dissimilarity. The number of singleton OTUs assigned to Eukaryota was over-estimated. Most of the reads in the singletons were a result of false positive amplifications by the universal primers. (DOCX) [file pone.0090053.s006.docx]

Table S5 Statistics of OTUs using 3’ reads of the amplicons at 3% dissimilarity

| E1,E2,I1 | %reads | No.OTUs | Singleton OTUs |
| --- | --- | --- | --- |
| Eukaryota | 95.8% | 97 | 64 |
| sponge | 92.9% | 5 | 0 |
| Bacteria | 3.4% | 108 | 67 |
| Archaea | 0.8% | 13 | 7 |
| Total reads | 14815 |  |  |
| I2 |  |  |  |
| Eukaryota | 42.2% | 30 | 21 |
| sponge | 37.2% | 3 | 1 |
| Bacteria | 56.5% | 150 | 93 |
| Archaea | 0.2% | 2 | 1 |
| Total reads | 1155 |  |  |

The number of singleton OTUs assigned to Eukaryota was over-estimated. Most of the reads in the singletons were a result of false positive amplifications by the universal primers.
